# Effect of Lymphocyte Subsets on Bone Density in Senile Osteoporosis: A Retrospective Study

**DOI:** 10.1155/2022/3337622

**Published:** 2022-10-26

**Authors:** Cong Peng, Zongwei Guo, Yue Zhao, Rui Li, Liang Wang, Wenping Gong

**Affiliations:** ^1^Tuberculosis Prevention and Control Key Laboratory/Beijing Key Laboratory of New Techniques of Tuberculosis Diagnosis and Treatment, Senior Department of Tuberculosis, The Eighth Medical Center of PLA General Hospital, Beijing 100091, China; ^2^Department of Geriatrics, The Eighth Medical Center of PLA General Hospital, Beijing 100091, China; ^3^Hebei North University, Zhangjiakou, 075000 Hebei, China; ^4^Institute of Respiratory and Critical Medicine/Beijing Key Laboratory of OTIR, The Eighth Medical Center of PLA General Hospital, Beijing 100091, China; ^5^Academy of Integrated Chinese and Western Medicine, Fujian University of Traditional Chinese Medicine, Fuzhou 350122, China

## Abstract

**Background:**

Several studies have shown that lymphocyte subsets can mediate the occurrence of osteoporosis (OP); however, the predictive ability of lymphocyte subsets in senile OP has not been elucidated.

**Purpose:**

To investigate the ability of lymphocyte subsets to predict senile osteoporosis (OP).

**Methods and Materials:**

This study included 44 patients with senile OP and 44 without OP. Dual-energy X-ray absorptiometry (DEXA) was used to determine bone mineral density (BMD). Flow cytometry was used to analyze the absolute counts of the lymphocyte subsets and cytokine levels. Finally, the correlation between BMD and lymphocyte subset counts in the two groups was analyzed.

**Results:**

There were no significant differences in age, sex, or weight between the OP and non-OP groups. The absolute counts of total T lymphocytes and CD8^+^ T lymphocytes in the OP group were significantly lower than those in the non-OP group. The levels of IFN-*γ* or TNF-*α* in the OP group were significantly higher or lower, respectively, than those in the non-OP group. PCA showed that age, BMI, total T lymphocytes, CD4^+^ T lymphocytes, CD8^+^ T lymphocytes, and B lymphocytes were the principal components of senile OP. The linear regression equation showed that BMD of the right femoral neck significantly decreased with a decline in CD8^+^ T lymphocyte counts.

**Conclusion:**

BMD decreased with a decrease in CD8^+^ T lymphocytes. The mechanism by which lower lymphocyte subsets lead to lower BMD may be related to abnormal bone metabolism caused by immune aging. Therefore, we considered that CD8^+^ T lymphocytes could be used to predict the incidence of senile OP.

## 1. Introduction

Senile osteoporosis (OP) is a disease of progressive loss of systemic bone mass caused by a variety of causes, characterized by low bone mass and destruction of bone structure. As bone strength decreases, the risk of fracture increases significantly in patients with senile OP [1]. Unlike menopausal OP, senile OP is more common among older adults. As the global population ages, the number of senile OP and associated fractures continues to increase worldwide [2]. A recently published large-scale study in China confirmed that the incidence of senile OP increased significantly with increasing age in both men and women aged > 55 years based on bone mineral density (BMD) detected using dual-energy X-ray absorptiometry (DEXA) [3]. This suggests that the aging population increases the demand for diagnosis, treatment, and prevention of senile OP and its complications. Meanwhile, body aging leads to a progressive decline in immune function [4]. Therefore, it is worth noting that aging of the immune system may further increase the incidence of senile OP.

Arron and Choi introduced the term “osteoimmunology” in 2000, emphasizing the two-way communication between the immune and skeletal systems [5]. For instance, the immune cell system or local activation can lead to the overexpression of immune factors, which leads to the dysregulation of the osteoprotegerin (OPG)/receptor activator for nuclear factor-*κβ* (RANK)/receptor activator for nuclear factor-*κβ* ligand (RANKL) system [6]. This regulatory mechanism is typical of osteoimmunology and mediates bone metabolism abnormalities. These data suggest that an aging-leading maladjusted immune system mistakenly regulates bone metabolism in patients with abnormal bone metabolism and senile OP.

Currently, senile OP diagnosis and prediction rely on DEXA, which has been used worldwide owing to its excellent accuracy, precision, and low radiation exposure [7, 8]. However, recent studies have shown that BMD-based fracture risk prediction underestimated the fracture risk in some populations, for example, in a population with diabetes [9]. In addition, the FRAX fracture risk prediction tool involving BMD underestimates the fracture risk in certain populations [10]. These data suggest that a better method is urgently needed to predict and evaluate BMD in patients with OP.

A growing number of studies have found that the ratio of lymphocytes to neutrophils, monocytes, and platelets has a particular predictive ability for senile OP [11–13], and circulating T lymphocyte subsets can predict bone morphology [14]. However, the predictive power of lymphatic subsets for senile OP has not been explored. In this study, we retrospectively analyzed the correlation between the basic situation, lymphocyte subsets, and BMD in patients with and without senile OP. This study is aimed at developing a new evaluation index for senile OP and its complications.

## 2. Material and Methods

### 2.1. Study Design and Ethics Statement

This retrospective study was performed at the Department of Geriatrics, Eighth Medical Center, PLA General Hospital. This study was approved by the Ethics Committee of the Eighth Medical Center of the PLA General Hospital (approval number: 202205311005) and conducted in accordance with the ethical standards of the Declaration of Helsinki.

### 2.2. Participants

This study included 88 elderly patients hospitalized in the Department of Geriatrics between December 10, 2021, and April 8, 2022. The diagnostic criteria were as follows: (1) definition of older adults (≥65 years) according to the internationally accepted definition of elderly population criteria [15] and (2) the diagnosis of senile OP according to “The Diagnosis of Osteoporosis” released by the World Health Organization (WHO) [16]. Participants with a BMD score ≤ −2.5, standard deviation (SD), were divided into the OP group, and volunteers with a BMD score > −2.5 SD were divided into the non-OP group. The exclusion criteria were as follows: (1) previous orthopedic diseases characterized by bone damage; (2) primary immune dysfunctional diseases, acquired immune deficiency, hematologic diseases, psychiatric disorders, severe infections, or allogeneic blood transfusion within three months; (3) patients with malignant tumors, such as lung cancer, liver cancer, major organ failure, or organ transplantation; (4) patients taking immunomodulatory agents and glucocorticoids within one year or other hormone supplementation treatment within one year; and (5) other diseases causing abnormal bone metabolism and immune regulation.

### 2.3. Clinical Characteristics of the Participants

General information on each participant was collected, including age, sex, height, weight, and BMI. Furthermore, we investigated the medical history of each participant, including hypertension, diabetes, coronary heart disease, malignancy, fracture, history of acute and critical illness, history of blood transfusion, and immune deficiency.

### 2.4. Absolute Count of Lymphocyte Subsets

Twenty microliters of lymphocyte subpopulation analysis reagent was added to the bottom of the absolute count tube (BD Biosciences, San Jose, CA, USA). These lymphocyte subpopulation analysis reagents included total T lymphocytes (CD3^+^), helper/inducer T lymphocytes (CD3^+^CD4^+^), suppressor/cytotoxic T lymphocytes (CD3^+^CD8^+^), B lymphocytes (CD3^−^CD19^+^), and natural killer cells (CD3^−^CD16^+^CD56^+^). Subsequently, 50 *μ*L of whole blood was drawn from a premixed EDTA anticoagulation tube (BD Biosciences, San Jose, CA, USA) and delivered to the bottom of the absolute counting tube using a reverse pipetting method. The tubes were shaken, mixed, and incubated for 15 min at room temperature to protect them from the light. Next, 450 *μ*L of hemolysin (BD Biosciences, San Jose, CA, USA) was added to each tube (FACS lysate: distilled water, 1 : 10). The samples were thoroughly mixed, incubated for 15 min at room temperature, and protected from light. The absolute number of lymphocyte subpopulations was determined using a FACS Canto Plus flow cytometer (BD Biosciences, San Jose, CA, USA). The CD4^+^/CD8^+^ T lymphocyte ratio was calculated based on helper/inducible T lymphocyte counts versus suppressor/cytotoxic T lymphocyte counts.

### 2.5. Cytokine Detection

Plasma was isolated from 20 OP and 20 non-OP patients. The concentrations of IFN-*γ*, TNF-*α*, IFN-*α*, IL-1*β*, IL-2, IL-4, IL-5, IL-6, IL-8, IL-10, IL-12P70, and IL-17 were detected using a multiple cytokine detection kit (RAISECARE, Qingdao, China) following our previous studies [17–19]. Briefly, 25 *μ*L of the experimental buffer was added to the sample tube. Next, 25 *μ*L of plasma, 25 *μ*L of detection antibody, and capture microsphere antibody were sequentially added to the sample tube. The sample tube was incubated for 2 h (400-500 r/min) at room temperature (25 ± 1°C) with shock in the dark. After that, 25 *μ*L SA-PE was added to the sample tube; the sample tube was incubated for 0.5 h (400-500 r/min) at room temperature (25 ± 1°C) with shock in darkness. Finally, wash buffer (500 *μ*L × 1) was added to the sample tube and vortexed for several seconds before centrifugation (300–500 g) for 5 min, and the liquid was slowly poured out from the sample tube and inverted onto absorbent paper. Using the FACS Canto Plus flow cytometer (BD, USA) requirements, 120 *μ*L of washing buffer was added to the sample tube, vortexed for 10 s to make the microsphere resuspended, and immediately run on the machine for detection. The detection data were imported into LEGEND Plex V8.0 software for analysis and acquisition.

### 2.6. Bone Density

The bone density of each participant was determined using a densitometer (HOLOGIC model: HOLOGIC Discovery Wi, Bedford, MA, USA). Senile OP diagnosis was defined according to internationally accepted criteria using the *T* score [16]. Senile OP was diagnosed when the *T* score was ≤-2.5, and other primary and secondary causes were excluded.

### 2.7. Statistical Analysis

GraphPad Prism 9.4.1 software (San Diego, CA, USA) and the Statistical Package for Social Sciences for Windows 25.0 (IBM, Armonk, NY, USA) were used for analysis. Continuous variables in the normal distribution were expressed as mean ± standard deviation (mean ± SD). Continuous variables in the abnormal distribution were expressed as quartiles (50% (25%-75%)). Discontinuous variables were expressed as numbers. In the end, continuous variables in the normal distribution of the two groups were analyzed with the unpaired *t*-test. Continuous variables in the abnormal distribution were analyzed by the Mann-Whitney *U* test. Discontinuous variables were analyzed by the chi-square test.

In addition, simple linear regression analysis was performed to evaluate the relationship between BMD levels and total T lymphocytes, CD4^+^ T lymphocytes, CD8^+^ T lymphocytes, NK cells, B cell counts, CD4^+^/CD8^+^ T lymphocyte ratio, age, sex, and height. Principal component analysis (PCA) was used to evaluate the effects of different indicators (age, BMI, absolute values of total T lymphocytes, CD4^+^ T lymphocytes, CD8^+^ T lymphocytes, NK cells, B lymphocytes, and CD4^+^/CD8^+^ T lymphocyte ratio) on senile OP. The principal components were selected based on eigenvalues > 1. Monte Carlo simulations were performed on the input data, and the eigenvalues of the principal components were calculated. Then, the eigenvalues of the input data were compared with the average values of the corresponding PCs in the simulations. The random seed, number of simulations, and percentile level were set to auto, 1000, and 95%, respectively.

## 3. Results

### 3.1. Participant Characteristics

In this study, 88 participants aged > 65 years old (77.24 ± 8.79) were divided into two groups: OP and non-OP groups (Table 1). Forty-four participants (76.77 ± 8.14 years) were included in the OP group based on BMD, including 25 males and 19 females. Furthermore, the same sample size of participants (77.7 ± 9.47 years) was enrolled in the non-OP group, including 19 males and 25 females.

The results of the age, height, weight, and BMI between the OP and non-OP groups were compared using the unpaired *t*-test or Mann-Whitney *U* test according to data normality, and discontinuous data of the gender composition between the two groups were compared using chi-square tests. The results showed that height (*P* = 0.02) and weight (*P* = 0.021) in the OP group were significantly lower than those in the non-OP group (Table 1). In contrast, age, BMI, and sex were not significantly different between the OP and non-OP groups (Table 1; *P* > 0.05).

### 3.2. Bone Density Differences

We compared BMD values between the OP and non-OP groups. The results showed that the total lumbar spine BMD, total left hip BMD, left femoral neck BMD, right hip BMD, and right femoral neck BMD were significantly higher in the non-OP group than in the OP group (*P* < 0.001, Table 2).

### 3.3. The Impact of Different Measurements on the Patients with Senile OP

**Figure 1:**
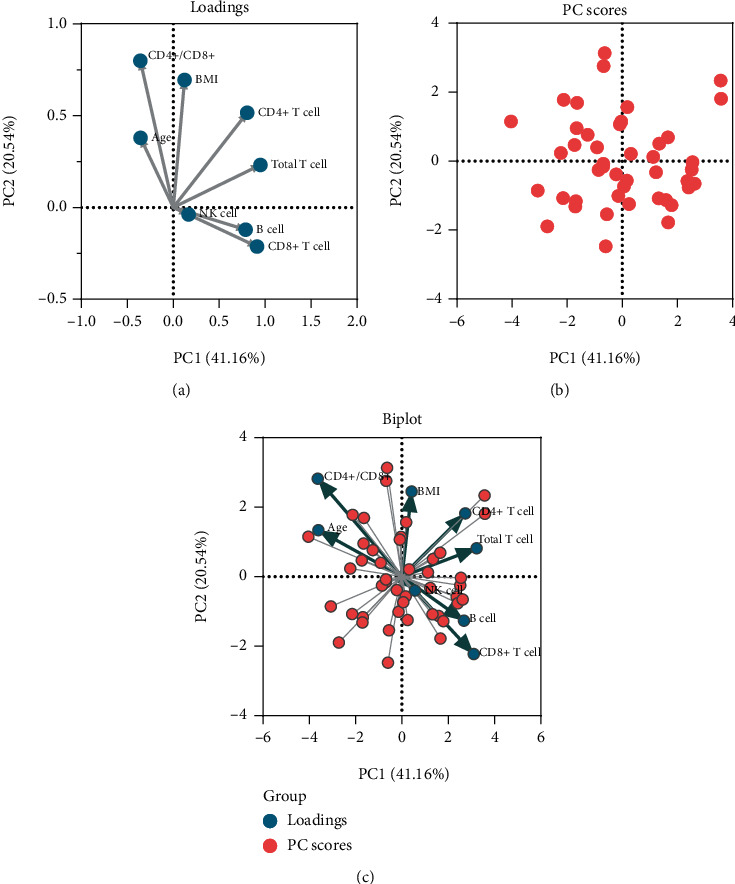
Principal component analysis (PCA) for different measurements on the patients with senile OP. The loading plot depicted the correlation of eight measurements and showed that age was associated with PC2. The absolute counts of CD8^+^ T lymphocytes were correlated with PC1 (a). The PC score plot showed that the data from 44 patients with OP were significant (b). Finally, a biplot was used to show both the loadings and PC scores (c).

The impact of different measurements on patients with senile OP (*n* = 44), including age, BMI, and the absolute number of total T lymphocytes, CD4^+^ T lymphocytes, CD8^+^ T lymphocytes, NK cells, B lymphocytes, and CD4^+^/CD8^+^ T lymphocyte ratios, was evaluated using PCA (Figure 1). Two potential PCs (PC1 eigenvalue = 3.293 and PC2 eigenvalue = 1.643) were selected according to eigenvalues > 1, and their cumulative variance contribution was 61.70% (PC1 41.16% and PC2 20.54%, Figure 1). The loading plot showed that age was associated with PC2, and the absolute counts of CD8^+^ T lymphocytes were correlated with PC1. The PC score plot showed that 44 patients had significant senile OP data.

### 3.4. Effect of Lymphocyte Subsets on Senile OP

**Figure 2:**
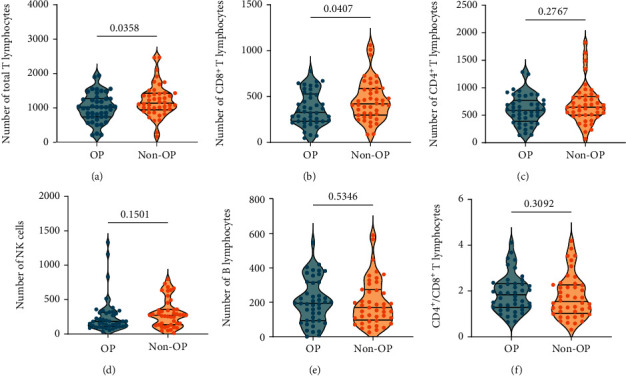
Comparison of the absolute counts of lymphocyte subsets between the OP and non-OP groups. The absolute counts of total T lymphocytes (a), CD8^+^ T lymphocytes (b), CD4^+^ T lymphocytes (c), NK cells (d), B cells (e), and CD4^+^/CD8^+^ T lymphocytes ratio (f) between the OP and non-OP groups were analyzed. Continuous variables in the normal distribution of the two groups were expressed as mean ± standard deviation (mean ± SD) and analyzed with the unpaired *t*-test. Continuous variables in the abnormal distribution were expressed as quartiles (50% (25%-75%)) and analyzed by the Mann-Whitney *U* test. *P* value < 0.05 was considered a significant difference.

To further explore the correlation between the absolute counts of lymphocyte subsets and senile OP, we compared the absolute counts of lymphocyte subsets in the OP and non-OP groups. The results suggested that the absolute counts of total T lymphocytes (*P* = 0.0358, Figure 2(a)) and CD8^+^ T lymphocytes (*P* = 0.0407, Figure 2(b)) were significantly lower in the OP group than in the non-OP group. In addition, there were no significant differences in the absolute counts of CD4^+^ T lymphocytes (*P* = 0.2767, Figure 2(c)), NK cells (*P* = 0.1501, Figure 2(d)), B cells (*P* = 0.5346, Figure 2(e)), and the CD4^+^/CD8^+^ T lymphocyte ratio (*P* = 0.3092, Figure 2(f)) between the OP and non-OP groups.

### 3.5. Linear Regression Analysis between Participants' BMD and Absolute Number of Lymphocytes

**Figure 3:**
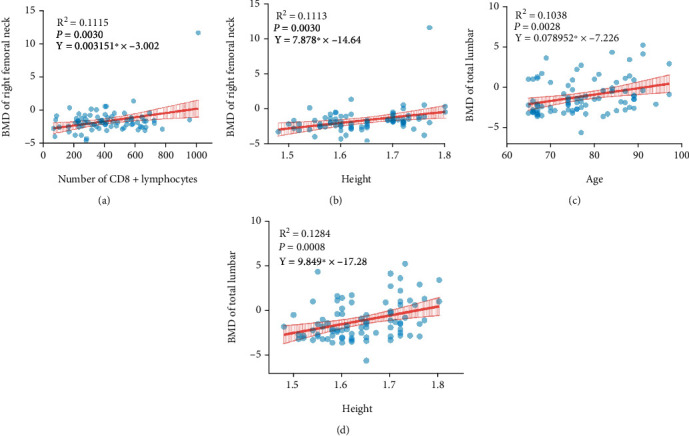
Linear regression analysis for evaluating the relationship among BMD levels, height, age, and absolute counts of lymphocyte subsets in patients with senile OP. Linear regression analysis of the relations between the right femoral neck BMD values and CD8^+^ T lymphocyte counts (a) or height (b) and the BMD of total lumbar and age (c) or height (d). *P* value < 0.05 was considered a significant linear relationship.

These results showed a clinical correlation between immune function and BMD in patients with senile OP. Therefore, we performed linear regression analysis to determine the relationship between BMD levels and absolute counts of lymphocyte subsets in patients with senile OP. We included two variables (age and height of the patient). Attempts were made to predict BMD values by analyzing more variables. Regression equations showed that the right femoral neck BMD values increased with increasing CD8^+^ T lymphocyte count (Figure 3(a), *P* = 0.0030, *R*^2^ = 0.1115, *Y* = 0.003151^∗^ *X* − 3.002) and height (Figure 3(b), *P* = 0.0030, *R*^2^ = 0.1113, *Y* = 7.878^∗^*X* − 14.64). Similarly, the BMD of the total lumbar area increased with age (Figure 3(c), *P* = 0.0028, *R*^2^ = 0.1038, *Y* = 0.078952 × *X* − 7.226) and height (Figure 3(d), *P* = 0.0008, *R*^2^ = 0.1284, *Y* = 9.849^∗^*X* − 17.28). There was no significant linear relationship between BMD values of total lumbar, left femoral neck, left hip, and right hip and lymphocyte subsets, including total T lymphocytes, CD4^+^ T lymphocytes, CD8^+^ T lymphocytes, NK cells, B cells, and the CD4^+^/CD8^+^ T lymphocyte ratio. Similarly, no significant linear relationship was observed between BMD values of the left and right femoral neck and total T lymphocytes, CD4^+^ T lymphocytes, NK cells, B cells, CD4^+^/CD8^+^ T lymphocyte ratio, height, and age.

### 3.6. Effect of Cytokines on Senile OP

**Figure 4:**
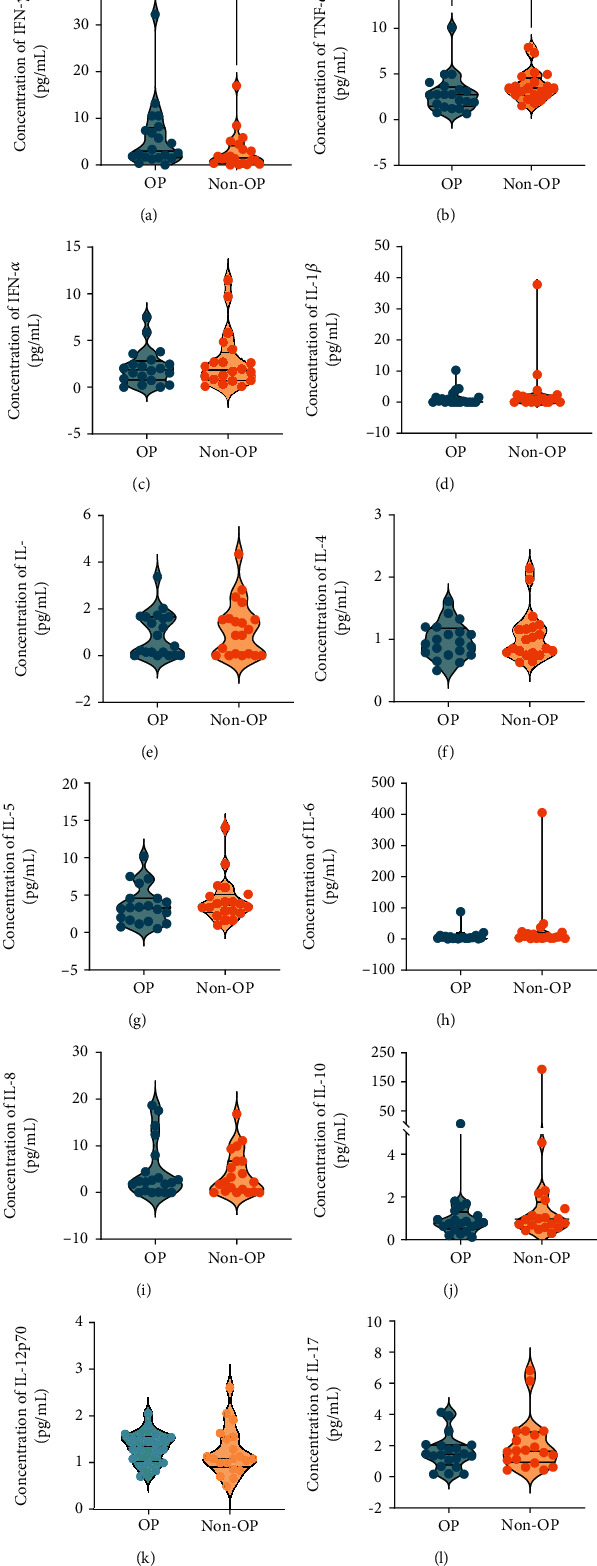
Comparison of the cytokines between the OP and non-OP groups. The concentration of IFN-*γ* (a), TNF-*α* (b), IFN-*α* (c), IL-1*β* (d), IL-2 (e), IL-4 (f), IL-5 (g), IL-6 (h), IL-8 (i), IL-10 (j), IL-12P70 (k), and IL-17 (l) between the OP (*n* = 20) and non-OP (*n* = 20) groups was analyzed with unpaired *t*-test or nonparametric test (Mann-Whitney *U* test) according to the normality. Continuous variables in the normal distribution of the two groups were expressed as mean ± standard deviation (mean ± SD) and analyzed with the unpaired *t*-test. Continuous variables in the abnormal distribution were expressed as quartiles (50% (25%-75%)) and analyzed by the Mann-Whitney *U* test. *P* value < 0.05 was considered a significant difference.

The results of lymphocyte subsets in senile OP suggested that total T lymphocytes and CD8^+^ T lymphocytes might be involved in the development of OP in our enrolled population. Herein, we detected the concentrations of IFN-*γ*, TNF-*α*, IFN-*α*, IL-1*β*, IL-2, IL-4, IL-5, IL-6, IL-8, IL-10, IL-12P70, and IL-17 in the OP (*n* = 20) and non-OP (*n* = 20) groups. Our results indicated that the IFN-*γ* level (*P* = 0.0436, Figure 4(a)) was significantly higher in the OP group than in the non-OP group, but the TNF-*α* level (*P* = 0.0479, Figure 4(b)) was significantly lower in the OP group than in the non-OP group. In addition, the levels of the remaining cytokines (IFN-*α*, IL-1*β*, IL-2, IL-4, IL-5, IL-6, IL-8, IL-10, IL-12P70, and IL-17) did not differ significantly between the two groups (Figures 4(c)–4(l)).

## 4. Discussion

In this study, we found significant differences in the T lymphocyte counts between senile OP and non-OP populations. T lymphocytes play an essential role in adaptive immune responses [18, 20, 21]. They are subjected to different environmental stimuli during activation (antigens, cytokines, etc.) and can differentiate into different subpopulations. Furthermore, a previous study indicated that T cell-deficient mice showed increased osteoclastogenesis and decreased bone mineral density, demonstrating that T cells play an essential role in preserving bone homeostasis in vivo [22]. Interestingly, other studies have confirmed that inactivated T helper (Th) cells inhibit osteoclast formation both in vitro and in vivo [23]. This may be related to the fact that Th cells do not secrete RANKL under steady-state conditions [24]. In contrast, under inflammatory conditions, T cell activation leads to enhanced production of RANKL (a member of the tumor necrosis factor superfamily) and TNF-*α*, promoting osteoclastogenesis, various inflammatory events, and subsequent bone loss under autoimmune conditions [25]. This is consistent with our experimental results; an increase in TNF-*α* may aggravate the occurrence of OP in the enrolled population. However, breaking the inflammatory cascade at any stage effectively reduces bone loss [26]. Furthermore, studies performed on ovariectomized (OVX) mice have shown that OVX does not cause bone trabeculae or bone cortex loss in T cell-deficient nude mice [27–31]. These observations prove that abnormal T lymphocyte levels may cause abnormalities in bone metabolism [32–34].

Total T lymphocytes consist of CD4^+^ T lymphocytes and CD8^+^ T lymphocytes. Therefore, the reduction in total T lymphocytes may be associated with lower CD4^+^ T lymphocytes and/or CD8^+^ T lymphocytes. Herein, we found no significant differences in the counts of CD4^+^ T lymphocytes between senile OP patients and non-OP patients. Much evidence indicates that the differentiation of CD4^+^ T lymphocytes into Type 1 T helper (Th1) and Th2 cells is influenced by environmental cytokines [18, 20]. Furthermore, it has been reported that a state of Th2 dominance is associated with senile OP [35, 36], which suggests that the levels of Th2-type cytokines, such as IL-4, IL-5, IL-6, and IL-10, increased, and Th1-type cytokines, such as IFN-*γ*, IL-2, and TNF-*α*, decreased in patients with senile OP. In our study, IFN-*γ* was reduced in the OP group, which is consistent with the results of the previous studies. However, TNF-*α*, as mentioned above, was increased in the OP group, which is probably due to the secretion of TNF-*α* by other immune cells. Immune cells in the body will differentiate and produce different types of cytokines under the influence of various factors and mediate infectious or noninfectious diseases [37]. We will further explore the reasons for the increase in TNF-*α* in the senile OP population in future mechanistic studies. Meanwhile, reduced secretion of Th1-type cytokines attenuated the activation and proliferation of CD8^+^ T lymphocytes, thereby reducing the number of CD8^+^ T lymphocytes. This might explain why the counts of CD8^+^ T lymphocytes were remarkably lower in senile OP patients than in non-OP patients in this study.

CD8^+^ T lymphocytes are a crucial component of the adaptive immune system and play an essential role in immune defense against intracellular pathogens, such as viruses, bacteria, and tumors [38–40]. It is well known that CD8^+^ T lymphocytes can differentiate into different effector cells, such as naïve CD8^+^ T cells, effector CD8^+^ T cells, effector memory CD8^+^ T cells, and central memory CD8^+^ T cells [41, 42]. Similarly, CD8^+^ T lymphocytes are also involved in bone metabolic activities, and CD8^+^ T lymphocytes inhibit osteoclast formation by secreting various soluble proteins (such as OPG) [43]. In recent years, CD8^+^ T lymphocytes have also been shown to protect the bone from metastasis under bone tumor loads [44]. The bone and immune system are functional units because they are located in their common ecological niche (osteoimmune system), which leads to their permanent interaction at various anatomical sites [45]. Thus, OP development is related to immune aging, which is regulated by an imbalance in the aging immune system. For example, our current study found that a decrease in the number of CD8^+^ T lymphocytes reduced their bone-protective ability. A reduction in the total number of T lymphocytes also mediates the osteoprotective mechanism of resting T cells.

The inflammatory response triggered by immune aging further stimulates the osteolytic activity of T lymphocytes [46], which is consistent with the results of this study. The regression equation showed that lower BMD values were associated with lower CD8^+^ T lymphocyte count. The decrease in CD8^+^ T lymphocytes may decrease BMD values of the left and right femoral necks. This again demonstrates a clear clinical correlation between lymphocyte subsets and bone. In clinical practice, we usually observe a significant increase in the prevalence of senile OP with increasing age. In contrast, our study showed that BMD increases with age and height. These differences may originate from the following factors: (1) the sample size of this study is too small; (2) the heterogeneity among these participants; (3) because all data sources come from manual measurements, operational errors are theoretically possible; and (4) the different measurement methods and test instruments may also contribute to the contradictory results. Therefore, we will expand the sample size in the future to further discuss the relationship between age and BMD.

To explore possible factors affecting BMD in patients with OP, we performed PCA on total T lymphocytes, CD4^+^ T lymphocytes, CD8^+^ T lymphocytes, CD4^+^/CD8^+^ T lymphocyte ratio, B cells, NK cells, age, and BMI. The results showed that age and CD8^+^ T lymphocyte count were the main constituents of the senile OP clinical profile. BMD is commonly used in the current diagnosis of bone metabolism abnormalities by defining the bone metabolism of a subject with a *T* score. It is an expression of BMD and is used for diagnostic classification according to the criteria established by the WHO for postmenopausal women or men aged 50 years or older [16]. A lower BMD represents a greater risk of fracture, with the risk of fracture doubling for every one standard deviation decrease in BMD [47]. However, as mentioned above, with the increase in the use of DEXA and FRAX, the deficiencies and shortcomings of their exposure also increase, mainly in terms of both diagnostic and predictive power. For example, senile OP is a different type of menopausal OP. As mentioned above, it is mainly reflected in the increase in incidence after the increase in age, unlike the sex difference in menopausal OP. This may be why there was no sex difference in the OP population in this study. It is also because of the sex difference in menopausal OP that the overall incidence of OP has a significant sex difference. Thus, more diverse evaluation methods are required to predict different types of OP. This study explored the correlation between lymphocyte count and BMD in patients with senile OP to identify more valuable indicators for evaluating bone metabolism and fracture risk.

However, this study has several limitations: (1) we did not include more bone metabolism indicators to assess bone metabolism from multiple perspectives, such as beta-isomer of the C-terminal telopeptide of type I collagen (*β*-CTX), which reflects osteoclast activity, and procollagen type I N-terminal propeptide (P1NP), which reflects osteoblast activity; (2) we did not collect abdominal and hip circumference data, which are essential to assess the relationships between the degree of skeletal stress and bone metabolism; (3) in order to avoid the influence of bone damage on the observed factors, patients with bone damage, such as fractures, were excluded in our study. However, in theory, whether fracture occurs has a particular effect on CD4^+^ T lymphocytes, CD8^+^ T lymphocytes, and the ratio of these two types of lymphocytes. This could also have an impact on the other indicators. Therefore, it is necessary to include different conditions of bone damage in further analyses, which will be improved in our subsequent study. (4) The sample size of this study was small, and these conclusions need to be verified in future studies with large sample sizes. (5) This study only involved the number of lymphocyte subsets but did not measure serum cytokine levels, which makes the analysis between lymphocytes and their secreted cytokines lack a strong basis. (6) Our study only included the Chinese Han population rather than other ethnic populations because there are differences in bone metabolism between Eastern and Western populations [48].

## 5. Conclusions

In summary, this study retrospectively assessed the potential relationship between lymphocyte subsets and bone metabolism in Han Chinese patients with senile OP. Our results showed that the absolute counts of total T lymphocytes and CD8^+^ T lymphocytes in patients with senile OP were significantly lower than those in the non-OP group, and the levels of IFN-*γ* and TNF-*α* were different between the OP and non-OP groups. These results suggest that a decrease in lymphocyte subsets might lead to BMD decline, suggesting that CD8^+^ T lymphocytes may have predictive power for BMD in the Chinese Han population with senile OP. We also found that age may be a relevant factor in the development of senile OP, according to PCA. Thus, the results of this study and the underlying mechanisms have substantial clinical conversion potential for the clinical practice of senile OP. This study provides novel insights into the prediction and evaluation of senile OP in the Han Chinese population, which is meaningful for preventing and controlling senile OP.

## Figures and Tables

**Table 1:** Clinical characteristics of the participants in this study.

Characteristics	Total^∗^ (*n* = 88)	Groups^∗^		*X* ^2^/*U*/*T* value	*P* value
		OP (*n* = 44)	Non-OP (*n* = 44)		
Age (years)	77.24 ± 8.79	76.77 ± 8.14	77.7 ± 9.47	0.495^a^	0.622
Male/female	44/44	25/19	19/25	1.636^b^	0.201
Height (m)	1.64 ± 0.08	1.62 (1.56, 1.695)	1.68 (1.595, 1.72)	-2.329^c^	0.020
Weight (kg)	62.53 ± 9.24	60.26 ± 6.92	60.81 ± 10.7	2.367^a^	0.021
BMI	23.12 ± 2.41	22.84 (21.64, 24.07)	22.885 (21.795, 24.455)	-0.442^c^	0.658

^∗^Values are shown as median (p25 and p75), mean ± SD, or number (percentage). ^a^The data of the two groups were compared with unpaired *t*-test. ^b^The data of the two groups were compared with chi-square test. ^c^The data of the two groups were compared with Mann-Whitney *U* test.

**Table 2:** Comparison of BMD values at different bone sites between OP and non-OP groups.

Place	Sample size (OP + non − OP)	Total^∗^	Groups^∗^		*U* value	*P* value
			OP	Non-OP		
Total lumbar spine	43 + 41	−1.299 ± 2.077	-2.8 (-3.2, -2.1)	-0.3 (-1.45, 1.2)	-6.087	<0.001
Left femoral neck	44 + 43	-1.9 (-2.55, -1.25)	-2.5 (-3, -2.4)	-1.2 (-1.75, -0.8)	-7.049	<0.001
Total right hip	44 + 43	-1.5 (-2.1, -0.6)	-2.1 (-2.6, -1.8)	-0.5 (-1.05, -0.2)	-7.055	<0.001
Right femoral neck	36 + 41	−1.674 ± 1.939	−2.267 ± 0.753	−0.666 ± 2.283	5.012	<0.001
Total left hip	36 + 41	-1.35 (-2.15, -0.55)	-2.1 (-2.5, -1.6)	-0.6 (-1.15, -0.05)	-6.192	<0.001

^∗^Values are shown as median (p25 and p75) and mean ± SD.

## Data Availability

All data generated or analyzed during this study are included in this published article.

## Abbreviations

BMD: Bone mineral density
BMI: Body mass index
CTL: Cytotoxic T lymphocytes
DEXA: Dual-energy X-ray absorptiometry
EDTA: Ethylenediaminetetraacetic acid
FACS: Fluorescence-activated cell sorting
(IGF)-1: Insulin-like growth factor
FRAX: Fracture risk assessment tool
OP: Osteoporosis
OPG: Osteoprotegerin
OVX: Ovariectomized
PCA: Principal component analysis
PCs: Principal component score
PC1: Principal component 1
PC2: Principal component 2
PTH: Parathyroid hormone
PGE2: Prostaglandin E2
RANK: Receptor activator for nuclear factor-*κβ*
RANKL: Receptor activator for nuclear factor-*κβ* ligand
TNF-*α*: Tumor necrosis factor-*α*
Wnt: Wingless and Int-1
*β*-CTX: Beta-isomer of the C-terminal telopeptide of type I collagen
P1NP: Procollagen type I N-terminal propeptide.

## References

[1] Bouvard B., Annweiler C., Legrand E. (2021). Osteoporosis in older adults. *Joint, Bone, Spine*.

[2] Curry S. J., Krist A. H., Owens D. K. (2018). Screening for osteoporosis to prevent fractures: US preventive services task force recommendation statement. *JAMA*.

[3] Zeng Q., Li N., Wang Q. (2019). The prevalence of osteoporosis in China, a nationwide, multicenter DXA survey. *Journal of Bone and Mineral Research*.

[4] Djukic M., Nau R., Sieber C. (2014). The ageing immune system. *Deutsche Medizinische Wochenschrift*.

[5] Arron J. R., Choi Y. (2000). Bone versus immune system. *Nature*.

[6] Lacey D. L., Timms E., Tan H. L., Kelley M. J., Boyle W. J. (1998). Osteoprotegerin ligand is a cytokine that regulates osteoclast differentiation and activation. *Cell*.

[7] Bergmann P. J. (2019). Change in bone density and reduction in fracture risk: a meta-regression of published trials. *Journal of Bone and Mineral Research*.

[8] Mazess R. B., Peppler W. W., Harrison J. E., McNeill K. G. (1981). Total body bone mineral and lean body mass by dual-photon absorptiometry. *Calcified Tissue International*.

[9] Willett M. J. R. M. V. D. W. C., Hu F. B. (2007). Systematic review of type 1 and type 2 diabetes mellitus and risk of fracture. *American Journal of Epidemiology*.

[10] Schwartz A. V., Vittinghoff E., Bauer D. C., Hillier T. A., Black D. M. (2011). Association of BMD and FRAX score with risk of fracture in older adults with type 2 diabetes. *JAMA*.

[11] Gao K., Zhu W., Liu W. (2019). The predictive role of monocyte-to-lymphocyte ratio in osteoporosis patient. *Medicine (Baltimore)*.

[12] Eroglu S., Karatas G. (2019). Platelet/lymphocyte ratio is an independent predictor for osteoporosis. *Saudi Medical Journal*.

[13] Huang C., Li S. (2016). Association of blood neutrophil lymphocyte ratio in the patients with postmenopausal osteoporosis. *Pakistan Journal of Medical Sciences*.

[14] Imai Y., Tsunenari T., Fukase M., Fujita T. (1990). Quantitative bone histomorphometry and circulating T lymphocyte subsets in postmenopausal osteoporosis. *Journal of Bone and Mineral Research*.

[15] Crews D. E., Zavotka S. (2006). Aging, disability, and frailty: implications for universal design. *Journal of Physiological Anthropology*.

[16] Kanis J. A., Rd M. L., Christiansen C., Johnston C. C., Khaltaev N. (1994). The diagnosis of osteoporosis. *Journal of Bone & Mineral Research*.

[17] Gong W., Liang Y., Mi J. (2021). A peptide-based vaccine ACP derived from antigens of *Mycobacterium tuberculosis* induced Th1 response but failed to enhance the protective efficacy of BCG in mice. *Indian Journal of Tuberculosis*.

[18] Gong W., Liang Y., Mi J. (2021). Peptides-based vaccine MP3RT induced protective immunity against *Mycobacterium tuberculosis* infection in a humanized mouse model. *Frontiers in Immunology*.

[19] Wang P., Xiong X., Jiao J. (2017). Th1 epitope peptides induce protective immunity against *Rickettsia rickettsii* infection in C3H/HeN mice. *Vaccine*.

[20] Gong W., Wu X. (2021). Differential diagnosis of latent tuberculosis infection and active tuberculosis: a key to a successful tuberculosis control strategy. *Frontiers in Microbiology*.

[21] Gong W., Liang Y., Wu X. (2018). The current status, challenges, and future developments of new tuberculosis vaccines. *Human Vaccines & Immunotherapeutics*.

[22] Yan L., Toraldo G., Li A., Yang X., Weitzmann M. N. (2007). B cells and T cells are critical for the preservation of bone homeostasis and attainment of peak bone mass in vivo. *Blood*.

[23] Toraldo G., Roggia C., Qian W. P., Pacifici R., Weitzmann M. N. (2003). IL-7 induces bone loss *in vivo* by induction of receptor activator of nuclear factor kappa B ligand and tumor necrosis factor alpha from T cells. *Proceedings of the National Academy of Sciences*.

[24] John V., Hock J. M., Short L. L., Glasebrook A. L., Galvin R. J. (1996). A role for CD8+ T lymphocytes in osteoclast differentiation in vitro. *Endocrinology*.

[25] Boyce B. F., Xing L. (2008). Functions of RANKL/RANK/OPG in bone modeling and remodeling. *Archives of Biochemistry and Biophysics*.

[26] Colucci S., Brunetti G., Rizzi R. (2004). T cells support osteoclastogenesis in an in vitro model derived from human multiple myeloma bone disease: the role of the OPG/TRAIL interaction. *Blood*.

[27] Faienza M. F., Ventura A., Marzano F., Cavallo L. (2013). Postmenopausal osteoporosis: the role of immune system cells. *Clinical & Developmental Immunology*.

[28] Roggia C., Gao Y., Cenci S. (2001). Up-regulation of TNF-producing T cells in the bone marrow: a key mechanism by which estrogen deficiency induces bone loss in vivo. *Proceedings of the National Academy of Sciences of the United States of America*.

[29] Yamaza T., Miura Y., Bi Y. (2008). Pharmacologic stem cell based intervention as a new approach to osteoporosis treatment in rodents. *PloS One*.

[30] Cenci S., Weitzmann M. N., Roggia C. (2000). Estrogen deficiency induces bone loss by enhancing T-cell production of TNF-alpha. *Journal of Clinical Investigation*.

[31] Gao Y., Qian W. P., Dark K. (2004). Estrogen prevents bone loss through transforming growth factor beta signaling in T cells. *Proceedings of the National Academy of Sciences of the United States of America*.

[32] Li J. Y., Tawfeek H., Bedi B. (2011). Ovariectomy disregulates osteoblast and osteoclast formation through the T-cell receptor CD40 ligand. *Proceedings of the National Academy of Sciences*.

[33] Grassi F., Tell G., Robbie-Ryan M. (2007). Oxidative stress causes bone loss in estrogen-deficient mice through enhanced bone marrow dendritic cell activation. *Proceedings of the National Academy of Sciences of the United States of America*.

[34] Moreland L., Bate G., Kirkpatrick P. (2006). Abatacept. *Nature Reviews Drug Discovery*.

[35] De Martinis M., Di Benedetto M. C., Mengoli L. P., Ginaldi L. (2006). Senile osteoporosis: is it an immune-mediated disease?. *Inflammation Research*.

[36] Ershler W. B., Harman S. M., Keller E. T. (1997). Immunologic aspects of osteoporosis. *Developmental and Comparative Immunology*.

[37] Wei R., Li P., Xue Y., Liu Y., Gong W., Zhao W. (2022). Impact of diabetes mellitus on the immunity of tuberculosis patients: a retrospective, cross-sectional study. *Risk Management and Healthcare Policy*.

[38] Gong W., Qi Y., Xiong X., Jiao J., Duan C., Wen B. (2015). *Rickettsia* rickettsiiouter membrane protein YbgF induces protective immunity in C3H/HeN mice. *Human Vaccines & Immunotherapeutics*.

[39] Gong W., Xiong X., Qi Y., Jiao J., Duan C., Wen B. (2014). Surface protein Adr2 of _Rickettsia rickettsii_ induced protective immunity against Rocky Mountain spotted fever in C3H/HeN mice. *Vaccine*.

[40] Zhang N., Bevan M. J. (2011). CD8^+^ T cells: foot soldiers of the immune system. *Immunity*.

[41] Reiser J., Banerjee A. (2016). Effector, memory, and dysfunctional CD8^+^ T cell fates in the antitumor immune response. *Journal of Immunology Research*.

[42] Gonzalez S. M., Taborda N. A., Rugeles M. T. (2017). Role of different subpopulations of CD8^+^ T cells during HIV exposure and infection. *Frontiers in Immunology*.

[43] Choi Y., Woo K. M., Ko S. H. (2001). Osteoclastogenesis is enhanced by activated B cells but suppressed by activated CD8^+^ T cells. *European Journal of Immunology*.

[44] Zhang K., Kim S., Cremasco V., Hirbe A. C., Faccio R. (2011). CD8^+^ T cells regulate bone tumor burden independent of osteoclast resorption. *Cancer Research*.

[45] Geusens P., Lems W. F. (2011). Osteoimmunology and osteoporosis. *Arthritis Research & Therapy*.

[46] Body J. J., Fernandez G., Lacroix M., Vandenbussche P., Content J. (1994). Regulation of lymphocyte calcitonin receptors by interleukin-1 and interleukin-6. *Calcified Tissue International*.

[47] Cheng X. G., Lowet G., Boonen S., Nicholson P. H., Van der Perre G., Dequeker J. (1998). Prediction of vertebral and femoral strength in vitro by bone mineral density measured at different skeletal sites. *Journal of Bone and Mineral Research*.

[48] Zhang Z., Ou Y., Sheng Z., Liao E. (2014). How to decide intervention thresholds based on FRAX in central south Chinese postmenopausal women. *Endocrine*.

